# Surround suppression maps in the cat primary visual cortex

**DOI:** 10.3389/fncir.2013.00078

**Published:** 2013-04-25

**Authors:** Matthieu P. Vanni, Christian Casanova

**Affiliations:** Laboratoire des Neurosciences de la Vision, École d'Optométrie, Université de MontréalMontréal, QC, Canada

**Keywords:** area 17, area 18, intrinsic signals, orientation selectivity, cortical map, receptive field

## Abstract

In the primary visual cortex and higher-order areas, it is well known that the stimulation of areas surrounding the classical receptive field of a neuron can inhibit its responses. In the primate area middle temporal (MT), this surround suppression was shown to be spatially organized into high and low suppression modules. However, such an organization has not been demonstrated yet in the primary visual cortex. Here, we used optical imaging of intrinsic signals to spatially evaluate surround suppression in the cat visual cortex. The magnitude of the response was measured in areas 17 and 18 for stimuli with different diameters, presented at different eccentricities. Delimited regions of the cortex were revealed by circumscribed stimulations of the visual field (“cortical response field”). Increasing the stimulus diameter increased the spread of cortical activation. In the cortical response field, the optimal stimulation diameter and the level of suppression were evaluated. Most pixels (≥3/4) exhibited surround suppression profiles. The optimal diameter, corresponding to a population of receptive fields, was smaller in area 17 (22°) than in area 18 (36°) in accordance with electrophysiological data. No difference in the suppression strength was observed between both areas (A17: 25%, A18: 21%). Further analysis of our data revealed the presence of surround modulation maps, organized in low and high suppression domains. We also developed a statistical method to confirm the existence of this cortical map and its neuronal origin. The organization for center/surround suppression observed here at the level of the primary visual cortex is similar to those found in higher order areas in primates (e.g., area MT) and could represent a strategy to optimize figure ground discrimination.

## Introduction

It is well established that the stimulation of areas beyond the classical receptive field can modulate the responses of neurons in the primary visual cortex (Gilbert, 1977; Allman et al., 1985; DeAngelis et al., 1994; Levitt and Lund, 1997; Sengpiel et al., 1997; Polat et al., 1998; Fitzpatrick, 2000; Akasaki et al., 2002; Cavanaugh et al., 2002a,b). In the case of surround suppression, the inhibition is maximal when the area surrounding the classical receptive field is activated with a stimulus presented at the neuron's preferred orientation (and optimal spatial frequency in case of gratings). The mechanisms subtending surround interactions are not totally understood but are likely to come from a combination of intracortical interactions, feedback signals from higher-level areas, and feed-forward inputs from the lateral geniculate nucleus (Das and Gilbert, 1995; Toth et al., 1996; Angelucci et al., 2002; Ozeki et al., 2004; Wielaard and Sajda, 2006; Durand et al., 2007; Naito et al., 2007).

Surround modulations were also reported in higher-order areas such as the middle temporal (MT) cortex of monkeys (Allman et al., 1985) and the lateral suprasylvian sulcus of cats (von Grunau and Frost, 1983). In area MT, motion-related surround suppression was reported to be spatially organized into high and low suppression modules (Born and Tootell, 1992; Born, 2000). Indeed, using full-field random-dot patterns and 2-deoxyglucose as a neuronal activity marker, Born (2000) obtained a mosaic of densely and lightly labeled patches of cortex in MT. Single cells recordings indicated that neurons located in lightly labeled spots were suppressed by full screen stimulations while the ones located in the dense patches were enhanced. These findings suggested the existence of domains for global and local integration in area MT. While clusters of neurons with high surround suppression exist in the cat primary visual cortex (Yao and Li, 2002), no such columnar organization for center/surround interactions has been reported.

The columnar organization of neurons sharing the same selectivity was proposed to be a strategy to optimize the processing in neural circuits by limiting the extent of connections (Durbin and Mitchison, 1990; Koulakov and Chklovskii, 2001; Chklovskii and Koulakov, 2004). Should a cortical organization for the surround suppression be present at the level of the primary visual cortex of cats, it would indicate that this interaction is optimized at a low level of hierarchy, in contrast to primates. Moreover, this map would be appended to the catalog of cortical maps already characterized in the cat primary visual cortex such as those for orientation (Grinvald et al., 1986), direction (Shmuel and Grinvald, 1996), spatial frequency (Issa et al., 2000), ocular dominance (Bonhoeffer et al., 1995), and binocular disparity (Kara and Boyd, 2009), and help to fully appreciate the complex functional organization of the cortex.

In the present study, we investigated the possibility that surround interactions in the primary visual cortex possess a modular organization using optical imaging of intrinsic signals. This method has been successfully used to reveal retinotopic, orientation, and direction maps in the cat primary visual cortex (Shmuel and Grinvald, 1996, 2000; Vanni et al., 2010a). It is sensitive enough that stimuli of restricted size can evoke significant optical responses over a delimited portion of cortex (i.e., the “cortical response field,” Das and Gilbert, 1995; Toth et al., 1996). Toth et al. (1996) further demonstrated, by comparing responses to localized and full-field stimuli, that center-surround interactions can also be quantified by optical imaging.

This study had two specific objectives. The first was to characterize in detail the suppressive interactions in areas 17 and 18 by presenting stimuli of increasing size at different eccentricities, thus over a broad range of receptive field sizes. The second was to determine whether a spatial organization for suppression exists in the cat primary visual cortex. Analysis of our data revealed the presence of a novel organization map in the cat primary visual cortex, where domains of high and low suppression are segregated in modules.

## Materials and methods

### Animal preparation

Female adult cats (*n* = 13) weighing between 2.45 and 3.95 kg were used in this study. Several of these animals were also used to collect data for other studies (Vanni et al., 2010a,b). All procedures were made in accordance with the guidelines of the Canadian Council for the Protection of Animals, and the experimental protocol was accepted by the Ethics Committee of the Université de Montréal. The cats were placed in a stereotaxic frame and artificially ventilated with a mixture of halothane (Fluothane®, 2% during surgery, 0.6–1% during recordings) in O_2_/N_2_O (30/70%). Muscular relaxation was obtained by the continuous injection of gallamine triethiodide (2%) infused with 5% dextrose in a lactated Ringer's injection solution. End-tidal CO_2_, blood pressure, blood oxygen saturation, core body temperature, electroencephalogram, and electrocardiogram were continuously monitored to evaluate the depth of anesthesia and the animal welfare. Pupils were dilated with atropine sulfate 1% (Isopto®) and the eyes were protected using contact lenses of appropriate refractive power. An antibiotic (Tribissen 24%, 0.125 mL/kg/day) was administered to prevent infections. Large craniotomies (see Villeneuve et al., 2009) were performed over the primary visual cortex including areas 17 and 18. The *dura mater* was removed to visualize the cortex. The recording chamber's frame was attached to the skull with dental cement and filled with silicone oil (Polydimethylsiloxane, 200® fluid, viscosity 350 cSt, Sigma-Aldrich, Inc.). At the end of each experiment, animals were killed by an injection of sodium pentobarbital (Euthanyl, 100 mg/kg).

### Acquisition and stimulation

The cortex was illuminated at 545 nm to reveal the vascular pattern of the cortical surface and at 630 nm to record intrinsic signals. Images were recorded with a 12 bits CCD camera (1M60, Dalsa, Colorado Springs, USA) fitted with a macroscopic lens (Nikon, AF Micro Nikkor, 60 mm, 1:2.8D). Visual stimuli were generated using a custom made software (STIMPlus) and presented on a 21-in computer screen placed 19 cm in front of the cat's eyes and subtending 120 × 90° of visual angle. Stimuli consisted of sine-wave gratings (0.05–0.6 c/deg., mean luminance = 25 cd/m^2^) displayed monocularly to the eye giving the strongest response and drifting at a temporal frequency of 2 or 4 Hz. The spatial and temporal frequency parameters were chosen to optimize activation and delimit borders between areas 17 and 18 in each animal tested (Villeneuve et al., 2009; Vanni et al., 2010a,b). Gratings moving in different directions (4 or 8 different values) and at varying sizes (from 2° diameter to full-screen stimulation) were presented pseudo-randomly. Each stimulus was presented during 8 s (sampling frequency = 0.6 Hz) and spaced by a 10 s interval during which the next stimulus was presented but remained still. This was used to reduce the unspecific transient response associated with the presentation of the stimuli that could reduce the specific response associated with the spatiotemporal parameters. One recording session generally lasted ~8 h (e.g., 35 trials × [6 diameters × 8 directions + 1 blank] × 18 s = ~30000 s). It is important to note that, despite the long duration of the recording period, no variation in the orientation maps was observed (data not shown). The blind spot was back projected on the computer screen with a light source and was used to determine the position of the *area centralis* (Bishop et al., 1962).

### Offline processing

The data was imported into Matlab (The Mathworks, Nattick, MA) for further analysis. Trials (average of 30–40 trials) were summed for the same-orientation-opposite-direction and band-pass filtered to remove low and high frequency noise which may alter the modular organization. Band-pass cut-offs were chosen according to the periodicity of the orientation domains as described in Villeneuve et al. (2009). Pixels associated with non-neuronal structures (e.g., large blood vessels) were extracted from the analysis. Note that the area of the visual field to be stimulated was chosen to correspond to a cortical representation with a low density of vasculature. Then, signal amplitude and preferred orientation were calculated by vector averaging of responses or orthogonal differential responses (Bonhoeffer and Grinvald, 1996; Shmuel and Grinvald, 1996). For each pixel, the profile of the responses as a function of stimulus size was fitted by a “ratio of Gaussian” (Sceniak et al., 2001; Cavanaugh et al., 2002a; Tailby et al., 2007). The function adapted from Tailby et al. (2007) is:

$${\text{ROG}}_{d}=\frac{k_{e}\text{erf}\left(\frac{d}{w_{e}}\right)}{1+k_{i}\text{erf}\left(\frac{d}{w_{i}}\right)}$$

where *d* is stimulus diameter, *k*_*e*_ and *w*_*e*_ are the gain and spatial extent of the excitation center mechanism respectively, *k*_*i*_ and *w*_*i*_ are the gain and spatial extent of the inhibition surround mechanism and erf is the error function. The diameter value attributed to full-screen stimulation for the fit was 90°. The fitting computations were the same as described in Villeneuve et al. (2009) for spatial frequency evaluation. Briefly, the values *k*_*e*_, *k*_*i*_, *w*_*e*_, and *w*_*i*_ were varied to minimize the error root mean square by simplex search method (Lagarias et al., 1998). For each pixel, the ROG profile was created with the fitted values *k*_*e*_, *k*_*i*_, *w*_*e*_, and *w*_*i*_ and the optimal diameter was defined as the stimulus diameter associated with the maximum of the fitted curve. The level of suppression was calculated from this fitted curve with the following equation:

$$\text{Suppression}=100\cdot \frac{M_{FS}-M_{OD}}{M_{OD}}$$

where *M*_*OD*_ and *M*_*FS*_ are the magnitude of the ROG function at optimal diameter and full-screen stimulation, respectively. To control possible inaccuracies introduced by the choice of a “ratio of Gaussian” function in our study, a subset of data was also fitted with other models, such as the “difference of Gaussians.” The choice of model did not strongly affect the suppression strengths measured (data not shown).

The limits of the cortical activation were determined by a method adapted from Villeneuve et al. (2009). Briefly, the differences between the preferred orientations at each stimulus size and at full-screen stimulation (reference map) were calculated. Only pixels having a difference less than 22.5° were considered reliable and part of the cortical activation. The cortical response field subject to quantification in areas 17 and 18 consisted of the cortical activations obtained with stimulus sizes of 8 and 12°, respectively. These two values were chosen because they represented the first which produced a significant activation in areas 17 and 18, respectively. Pinwheel orientation locations, high and low optimal diameters and suppression region borders were determined by the 2D-gradient method described in Vanni et al. (2010a). The 2-D gradient is low when computed on homogeneous parts of the map (e.g., orientation domains pixels) and high, close to non-homogeneous regions (e.g., pinwheels).

### Shuffle analysis and statistics

The existence of functional maps has sometimes been questioned because of the possible contribution of blood vessels and cerebral fluctuations in the responses (Sirovich and Uglesich, 2004). To resolve this issue, we developed a method using shuffle computation to confirm that surround suppression maps came from a neuronal origin and not from cerebral and blood fluctuations. This method involved splitting trials of experiments into two subsets of trials, one with the correct sequence and the other shuffled. Since this reduction in trial number reduced the signal to noise ratio, we could successfully use the shuffle computation in some experiments only.

Considering the large number of pixels of each map, the statistical inference (p) obtained with cross-correlation is not valid and will not be shown. However, the *r*-values were exploited. To confirm that surround suppression maps came from a neuronal origin, 50% of trials were randomly chosen to create an optimal diameter map and the 50% remaining, to create a second. This procedure was repeated 20 times to give a distribution of *r*-values of cross-correlation from each pair of maps. This first distribution of *r*-values was compared to a second from the same dataset in which sizes' values were mixed to remove the surround contribution. Consequently, optimal diameter values calculated from these second shuffled datasets resulted only from cortical fluctuations. Then, paired *t*-test between these two *r*-values distribution were used to infer or not the hypothesis of a surround contribution on maps with statistical conclusion. The same procedure was applied for suppression strength maps.

The shuffle computation was also used to calculate interactions between orientations and both optimal diameter and suppression values. The *r*-values of cross-correlations between 2D-gradient of preferred orientation and optimal diameter maps were computed in 16 independent experiments. These 16 experiments were chosen out of a total of 31 because the same stimulus sizes and orientations were used to calculate optimal diameter values in area 18. This first distribution of *r*-values was compared to a second from the same dataset in which sizes' values had been mixed. No correlations were expected to be found between the optimal diameter maps from shuffled stimuli size and 2D-gradient of preferred orientation maps. To test the hypothesis of a spatial interaction between optimal diameter and 2D-gradient of preferred orientation maps, a paired *t*-test between these two *r*-values distribution was performed. The same procedure was applied for spatial interactions of 2D-gradient of preferred orientation with suppression strength maps and 2D-gradient of optimal diameter and suppression strength maps.

## Results

### Retinotopic maps and cortical response fields

**Figure 1:**
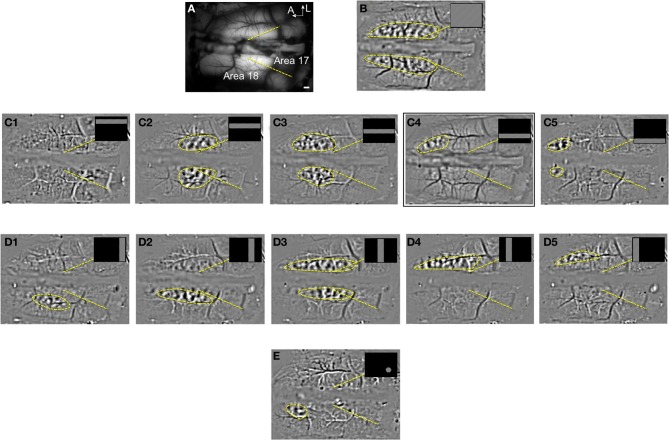
**Retinotopic map and cortical response field in area 18. (A)** Vasculature pattern of the visual cortex obtained under 546 nm illumination. Yellow dotted lines indicate the borders between areas 17 and 18. **(B)** Differential map evoked by a full-screen grating (4 Hz, 0.15 cpd). **(C)** Differential maps evoked by gratings confined in large horizontal bars (width of 12°) placed at different elevations (+5, −7, −19, −31, and −43°). Yellow dotted circular regions indicate the cortical response fields. **(D)** Differential maps evoked by gratings confined in 12° vertical bars positioned at different azimuths (+20, +10, 0, −10, and −20°). **(E)** Differential map evoked by gratings confined in a 12° window at −27 and +20° of elevation and azimuth respectively. Scale bar = 1 mm.

All activation zones in the present study were retinotopically defined. Figure 1 shows an example of retinotopic mapping in area 18 for animal case #1. In a large region of interest (panel A), orientation domains evoked by a full-field grating were clearly visible in the anterior part of the differential map (panel B). Confining the grating in horizontal bars at different spatial positions revealed the retinotopy along elevation (panel C). As described by others, the main part of the primary visual cortex accessible for imaging corresponded to regions representing the central and lower parts of the visual field (Tusa et al., 1978, 1979; Vanni et al., 2010b). A displacement of the retinotopic activation along elevation was seen in the anteroposterior axis, with the lower part of the visual field represented in the anterior part of the visual cortex. Confining the grating in vertical bars placed at different positions revealed the retinotopy along the azimuth (panel D). Finally, by restricting the grating in a circular window at a specific position of the visual field, it was possible to activate a limited region of the cortex, namely the “cortical response field,” in accordance to the retinotopic map (panel E). In maps presented thereafter, positions of the centers of stimuli were chosen to evoke cortical response within the limits of the craniotomy in zones exempt of large blood vessels. In the following sections, the activation in cortical response field is further explored as a function of stimulus size and eccentricity in the cortical area (17 or 18).

### Example of responses in area 18 as a function of stimulus size

**Figure 2:**
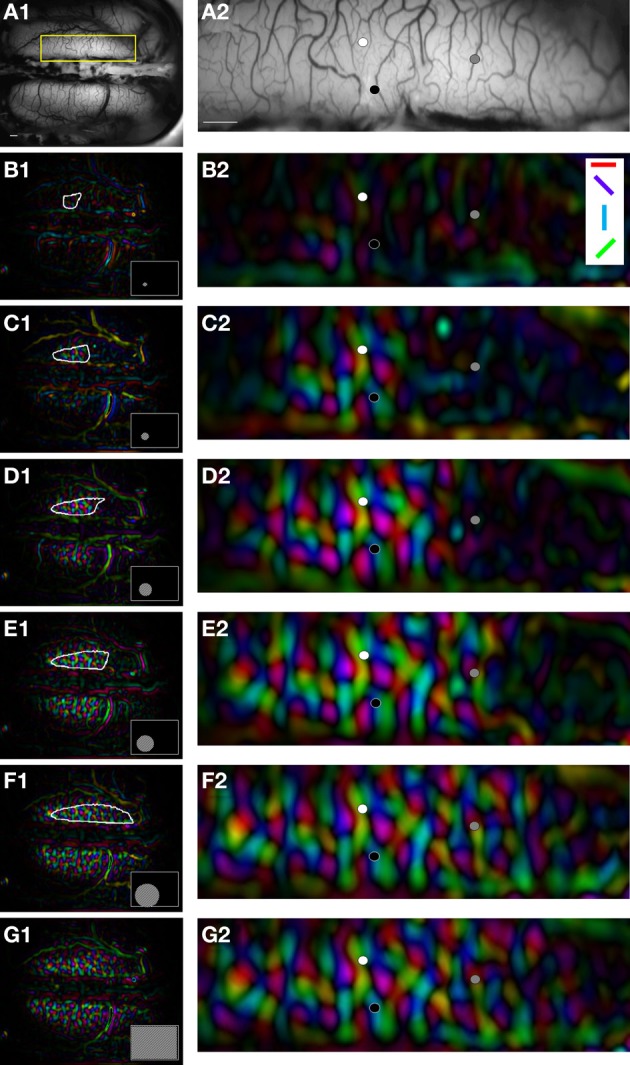
**Spread of activation as a function of the size in area 18. (A)** Vasculature pattern of the visual cortex. Panel **(A2)** shows a magnified view of the region of interest (yellow box) in panel **(A1)**. **(B–G)** Orientation maps evoked by different stimulus sizes (gratings: 0.15 cpd at 4 Hz, windows of 6, 12, 18, 24, and 40° and full-screen stimulation). Stimuli were placed at −22 and −6° of elevation, and azimuth. Colors designate preferred orientations [symbols in white box, panel **(B2)**] and brightness corresponds to signal magnitude. White outlines in panels **(B1–F1)** indicate the spread of activation for each stimulus size (see Methods). Black, gray, and white symbols in panels **(A2–G2)** represent the location of the measures shown in Figure 3. Scale bars = 1 mm.

Figure 2 shows the extent of the activation zone as a function of the stimulus size in area 18 of animal case #2. Orientation maps in area 18 (panel A) are shown for six stimulus sizes (panels B–G). The cortical response field borders are indicated by a white outline and increased with stimulus size: for small circles (panels B,C, 6 and 12°), the activation was confined in an area of some millimeters in the anterior and right part of the cortex. For larger circles (panels D–G, >18°), the activation became bilateral and encompassed the posterior part of area 18. The quantitative evaluation of the surface, length and width of the cortical response fields was not possible because, in most experiments, the boundaries of cortical response fields were not visible within the recording chamber. Nevertheless, we noticed in the current example and in most experiments that the surface of activation decreased with eccentricity, as expected from the reduction of the cortical magnification factor.

**Figure 3:**
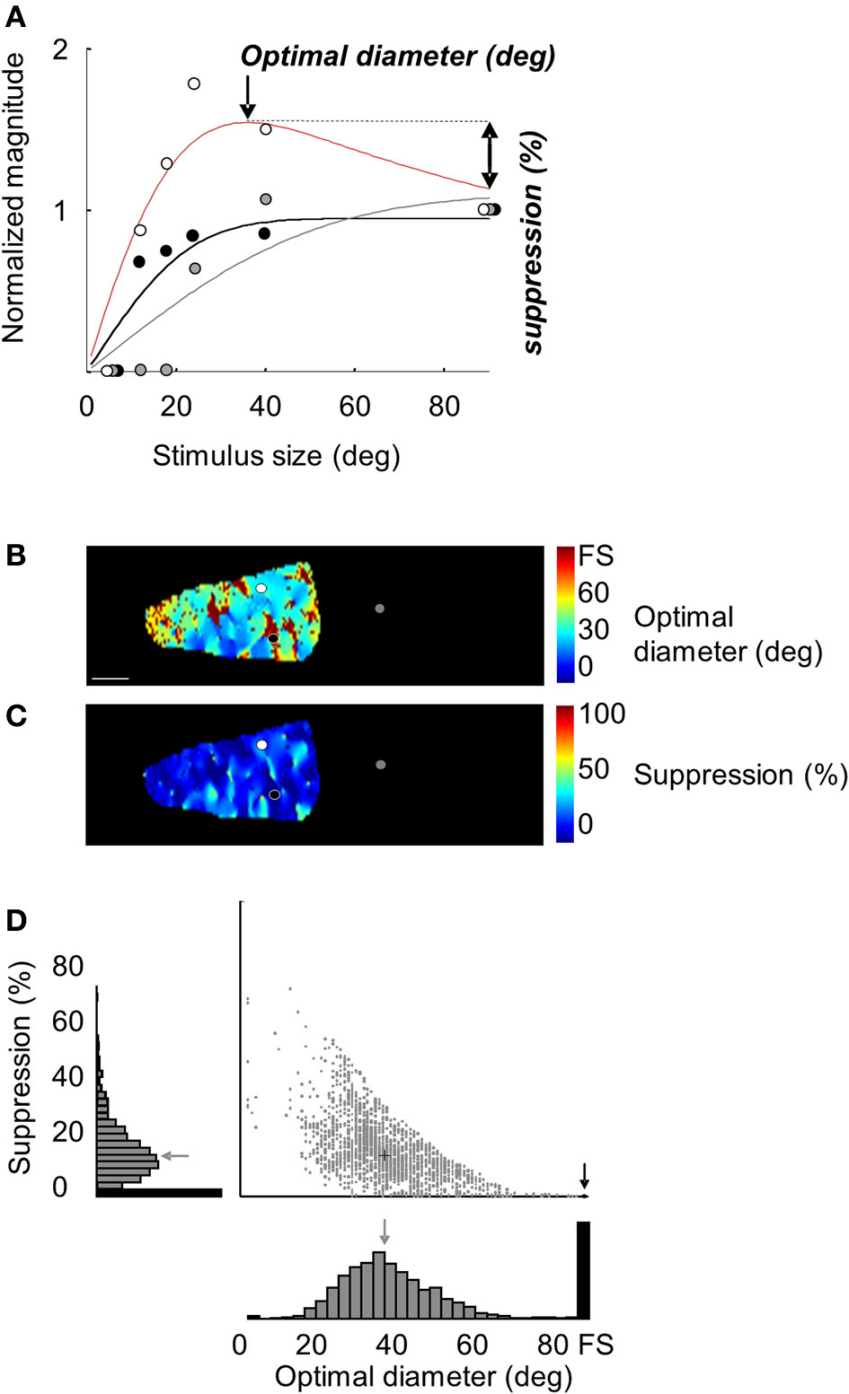
**Optimal diameters and suppression strength maps in area 18. (A)** Normalized magnitude values (symbols) as a function of stimulus size in the three locations showed in Figure 2 (black, gray, and white symbols). The lines are fits to the ROG function described in the Methods section. The optimal diameter and suppression strength values computed from the red line are 36° and 20%, respectively. The optimal diameter computed from the black curve was a “full field” without any suppression (0%). **(B)** Optimal diameters map in the ROI shown in Figure 2, panel 2. Black and white symbols are the location where measures shown in panel **(A)** were taken. **(C)** Suppression strength map. **(D)** Scatter plot and histograms of the distribution of optimal diameters and suppressions strength in the cortical response field. The gray arrows and the cross represent mean values. Gray bars in histograms and dots in the scatter plot correspond to pixels which exhibit a suppression profile. Black bars and the black circle correspond to pixels without suppression. FS, full screen. Scale bar = 1 mm.

While the limits of the cortical response field could not be quantitatively estimated, the orientation vectors could be measured with confidence. When examining the signal amplitude as a function of stimulus size, we identified domains in the cortical response fields that responded very differently to stimulus size increments. Some parts of the cortical response field were characterized by an absence of surround suppression, as their maximal response was obtained at full field stimulation (Figure 3A, black circles). Other cortical positions displayed surround suppression profiles, where the maximal response was obtained for a given stimulus size and where subsequent stimulus size increases caused a decrease in the signal amplitude (white circles). Outside the cortical response field, the signal magnitude was null for the smallest sizes because there was no cortical activation (gray circles). To further explore the characteristics of the responses to varying stimulus sizes at every locations of the cortical response field, the amplitude of the signal was fitted by the ROG function described in the Methods section.

For each pixel of the experiment shown in Figure 2, optimal diameters and suppression strength values were measured from the fitted curves to reveal corresponding cortical maps. Panels B,C display the optimal diameter and suppression strength maps in the cortical response field observed for a 12° stimulus size. For 15% of the pixels, optimal diameters consisted of the full field stimulation (brown pixels, panel B) without any suppression (deep blue pixels, panel C). Similar results were observed when signal amplitude was calculated from the amplitude of the orientation tuning curve (data not shown). Panel D displays the distribution of optimal diameters and suppression strength values in the cortical response field. For the pixels with surround suppression, the mean optimal diameter was 38° and the mean suppression strength was 15%.

### Example of responses in area 17 as a function of stimulus size

**Figure 4:**
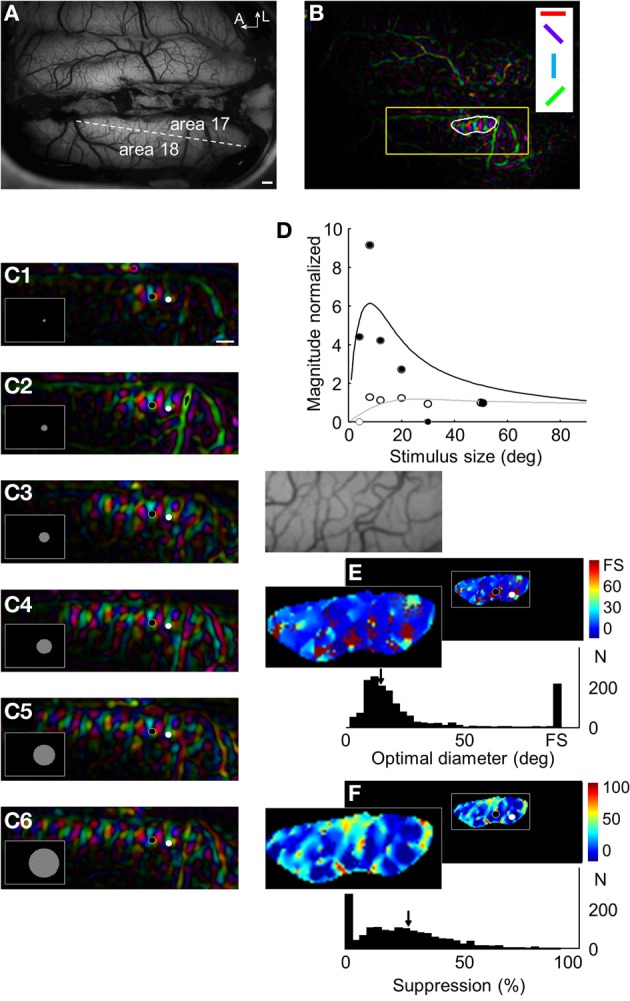
**Responses in area 17 to increasing stimulus sizes. (A)** Anatomical image of the visual cortex. The dotted line indicates the border between areas 17 and 18. **(B)** Orientation maps evoked by a grating (0.5 cpd at 2 Hz) in a 8° size window centered at −5° of elevation and +10° along the azimuth. The white outline identifies the cortical response field. **(C)** Magnified orientation maps within the region indicated by a yellow box in panel **(B)** for six stimulus sizes (4, 8, 12, 20, 30, and 50°). **(D)** Magnitude normalized (symbols) and fit (lines) in the two locations indicated in panel **(C)** (black and white symbols). **(E)** Optimal diameters map and distribution of values. The magnification of the anatomical image corresponding to the region of interest is presented below. **(F)** Suppression strength maps and distribution of values. In **(E)** and **(F)**, arrows represent mean values. Scale bars = 1 mm.

Increasing the stimulus size also yielded an increase of the cortical response field in area 17, and likewise, the magnitude response profile allowed to reveal suppression strength and optimal diameter values. Figure 4 displays the results obtained in animal case #3. Panel B shows a clear orientation map in area 17 (panel A) evoked by an 8° stimulus size. The responses for six increasing stimulus sizes are shown in panel C. As for area 18, the activation area increased with stimulus size. However, responses could be detected for smaller sizes (e.g., note the strong activation for 4 and 8° in panels C1,C2 in comparison to the weak activation in area 18 for a 6° stimulus size in Figure 2, panel B). The relationship between response magnitude and stimulus size in two locations within the cortical response field is presented in panel D. As in area 18, some locations had a maximum response for full field stimulation (white symbols) and others for smaller sizes (black symbols). Optimal diameter and suppression strength were then calculated for every pixel and shown in panels E,F, respectively. A structure similar to that in area 18 was observed: for 14% of the pixels, optimal diameters consisted on the full field stimulation without any suppression. For the remaining pixels, the mean optimal diameter (17°) was smaller than that found in area 18 and the mean suppression strength value was 27%.

### Interaction between optimal diameters, suppression strength values, and eccentricity

**Figure 5:**
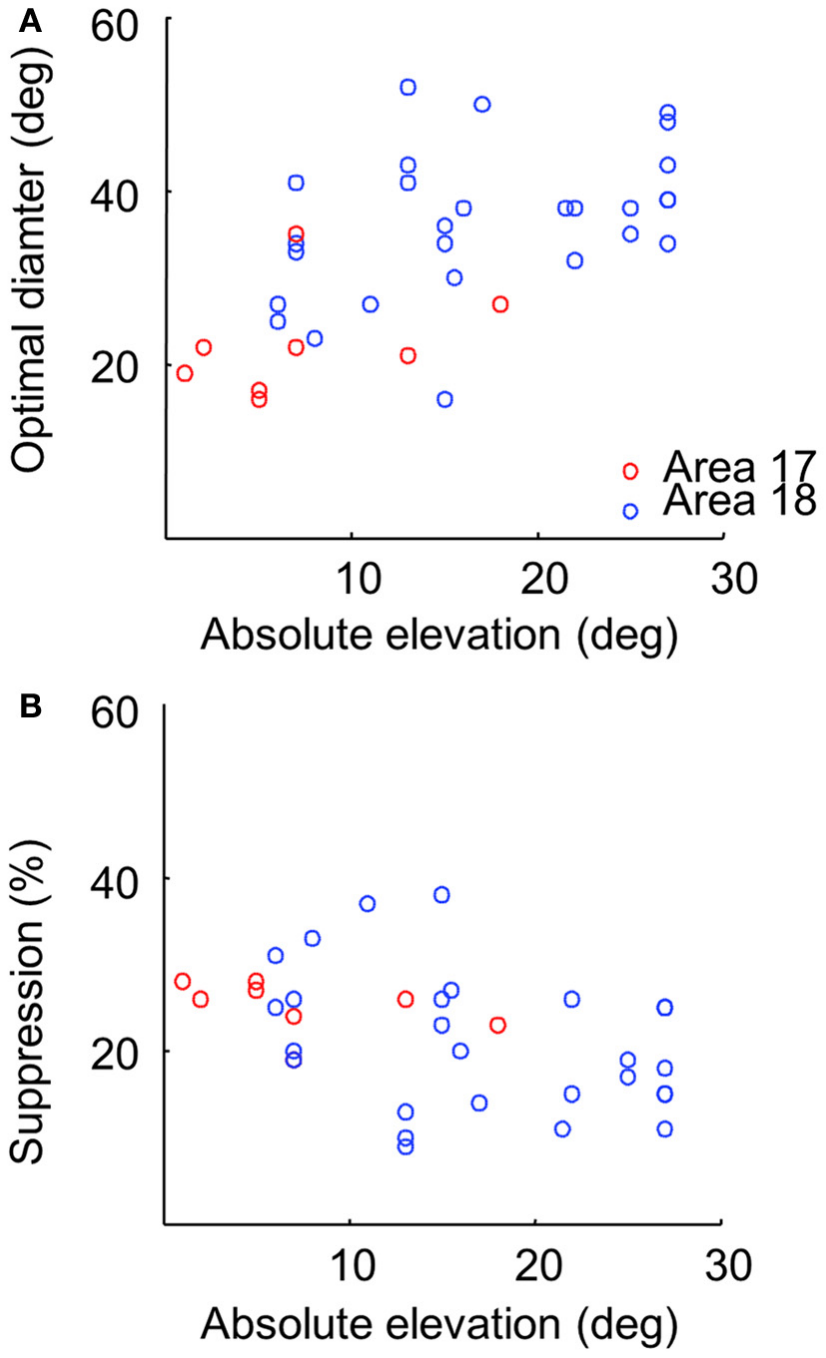
**Comparison of values as a function of brain areas and eccentricities. (A)** Scatter plot between optimal diameter values and absolute elevation in areas 17 (red symbols, correlation coefficient: *r* = 0.43, *p* = 0.248), 18 (blue symbols, *r* = 0.3, *p* = 0.105), and both 17 and 18: *r* = 0.5 (*p* = 0.001). **(B)** Scatter plot between suppression strength values and absolute elevation [area 17: *r* = −0.51 (*p* = 0.165), area 18: *r* = −0.29 (*p* = 0.12), area 17 + 18: *r* = −0.37 (*p* = 0.02)].

To calculate population values and reveal the relationships between receptive field sizes, optimal diameters and suppression strength values, we compiled the data from 36 independent tests measured at different eccentricities in areas 17 and 18. Analyses of our data indicate that the (surface of) domains showing no surround suppression are larger in area 18 than in area 17. Indeed, in area 18, 26.6 ± 10.0% (*n* = 28) of total pixels in the cortical response fields had their optimal stimulus size at full field stimulation (with no surround suppression), whereas the proportion for area 17 was of 17.1 ± 5.9% (*n* = 8) (*p* = 0.016, Student two-tailed *t*-test). For pixels which exhibited suppression, optimal diameters were significantly smaller in area 17 than in 18 (22.4 ± 6.1 and 36.1 ± 8.5°, respectively; *p* < 0.001, Student one-tailed *t*-test, Figure 5A). By contrast, no difference was observed in suppression strength values (area 17: 25.1 ± 3.0%, area 18: 21.4 ± 8.1%, *p* = 0.207, Figure 5B).

It has been shown that receptive field size increase with eccentricity (Tusa et al., 1978, 1979). We determined whether this observation can be confirmed with optical signals by measuring optimal diameters as a function of the eccentricity of stimulation. Panel (A) of Figure 5 shows that optimal diameter values tended to increase with elevation, in agreement with the established relation between receptive field size and eccentricity in mammals with a foveal representation. A weak but opposite tendency was also observed between suppression strength and eccentricity (panel B).

### Control of the neuronal origin of optimal diameters and suppression strength maps

**Figure 6:**
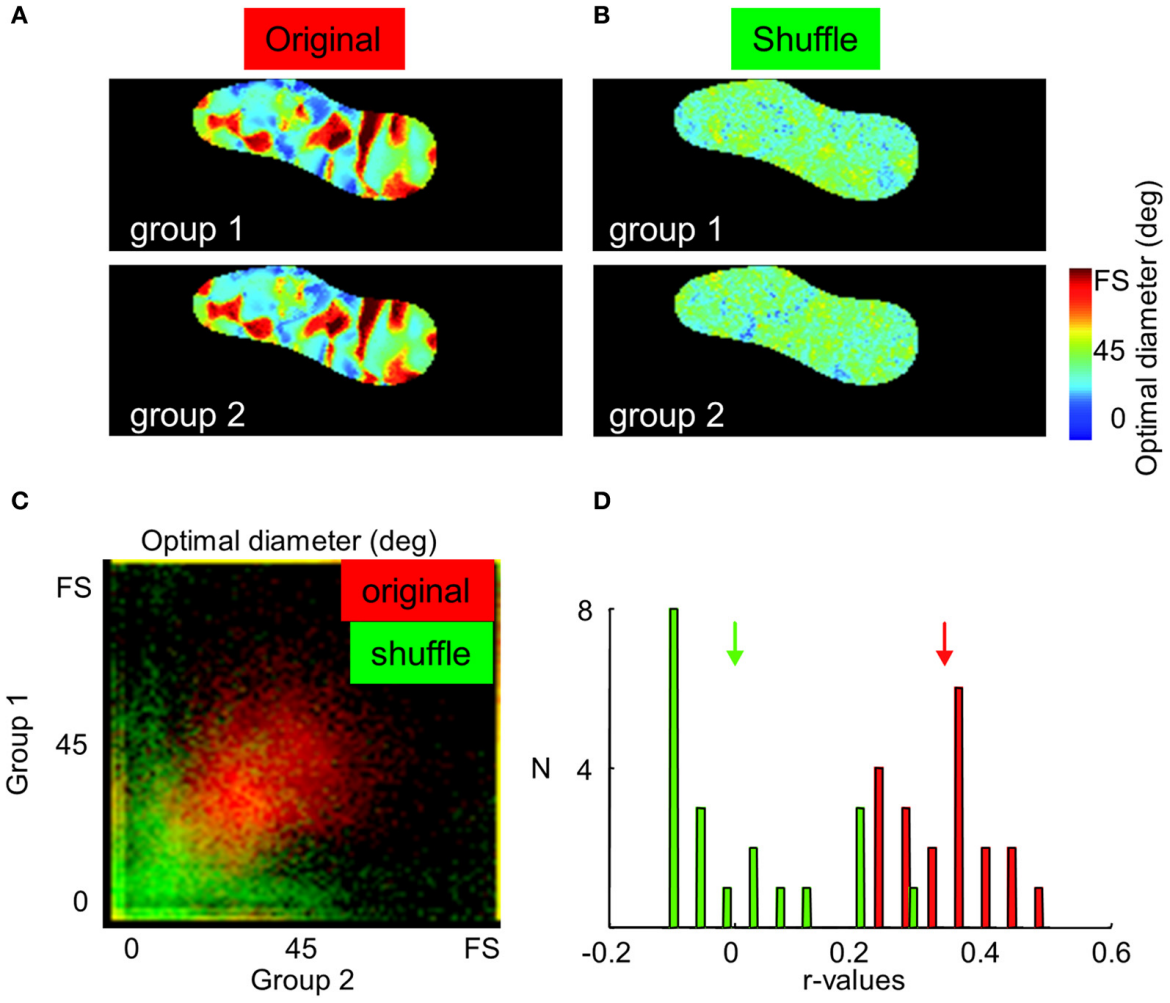
**Neuronal origin of optimal diameters and suppression strength maps. (A,B)** Average of 20 optimal diameter maps construct from half of the trials randomly chosen at each iteration in normal and shuffle conditions, respectively. Twenty trials were used to build each optimal diameter map. **(C)** Distribution of optimal diameters in every pixels of the map for the 20 iterations in normal (red) and shuffle (green) conditions. **(D)** Distribution of correlation values in these 20 iterations in normal (red) and shuffle (green) conditions. Arrows indicate mean values.

One may raise the possibility that surround maps originate mainly from noise and not from specific neuronal responses. In contrast to responses from a neuronal origin, noise signals are expected to be fully random across repetitions. Therefore, if an experiment is divided into two sub-experiments, the two sub-maps should be identical if they are built from neuronal signals. This last assumption is verified in Figure 6 from animal case #4. Panel A displays an average of 20 optimal diameter maps constructed from half of the trials randomly chosen at each iteration (see Methods). The two maps are identical. In contrast, no map can be seen in the shuffle condition (panel B, see Methods). Panel C displays the distribution of optimal diameter for every pixel of the map for the 20 iterations in normal (red) and shuffle (green) conditions while panel D presents the distribution of correlation values in these 20 iterations. A strong correlation was observed not only between optimal diameter maps (correlation between normal maps: 0.34 ± 0.08, and “stimulus size shuffled” maps: 0.00 ± 0.12, *p* < 0.001) but also between suppression strength maps (data not shown, normal maps: 0.35 ± 0.13, shuffle maps: 0.04 ± 0.20, *p* < 0.001).

### Spatial organization

**Figure 7:**
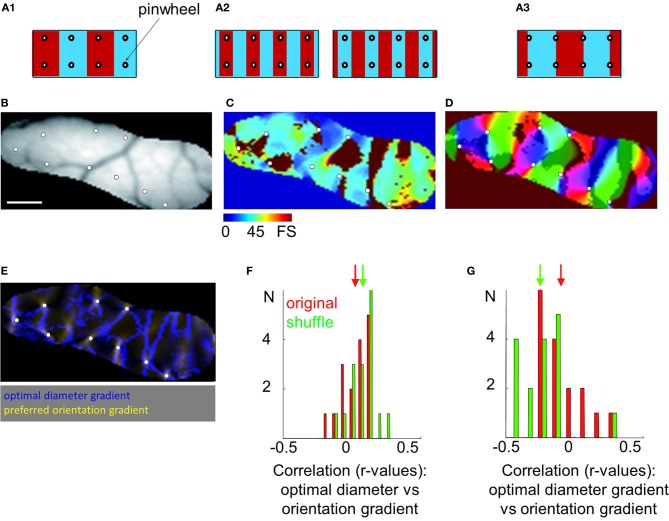
**Relationship between surround suppression and orientation maps. (A)** Different patterns of organization between orientation and optimal diameter/suppression strength maps are proposed. Blue and red bars represent high and low surround suppression domains, respectively. White circles are the location of pinwheels in the orientation map. They are easily identifiable by computing the 2D gradient of the preferred orientation map. **(B)** Vasculature pattern in the region of interest of the visual cortex. Scale bar = 1 mm. **(C)** Optimal diameter map. **(D)** Orientation map evoked by a grating (0.15 cpd at 4 Hz) presented in a 12° window. Bright and dark regions represent the superimposed locations of high and low surround suppression domains, respectively. **(E)** 2D-gradient of preferred orientation (yellow) superimposed on 2D-gradient of optimal diameter maps (blue). **(F)** Distribution of correlation values between 2D-gradient of preferred orientation and optimal diameter in 16 independent experiments (in red) and in a shuffle condition (in green). **(G)** Distribution of correlation values between 2D-gradient of preferred orientation and 2D-gradient of optimal diameter. In **(F)** and **(G)**, arrows indicate mean values.

We investigated whether there are interactions between surround suppression and preferred orientation maps throughout the surface of the cortex (Figure 7). Preferred orientation maps are organized in periodic domains of iso-orientations and singularities (i.e., pinwheels, see white circle in panel A). Relationships between surround suppression and preferred orientation maps could follow different patterns, illustrated in panel A. An organization with uniformity of coverage would have values of each parameter homogeneously represented in each position in the visual space. When this scenario is applied to the relationship between preferred orientation and ocular dominance parameters, pinwheels of preferred orientation map are generally uniformly distributed in the center of each functional domains of other maps [e.g., right or left eyes, panel A1 (Swindale, 1998, 2000, 2001)]. Another possibility could be that one value of a parameter is more represented in the proximity of pinwheels (e.g., high or low suppression strength, panel A2). A final option would be that pinwheels are only present in transitions between two opposite values of a parameter (e.g., transition between high to low suppression strength, panel A3).

A portion of area 18 in animal case #4 and the associated optimal diameters map are shown in panels B,C. The orientation map on which the optimal diameters map (bright and dark regions) was superimposed is presented in panel D. White symbols indicate the position of the pinwheels, which are mostly present at the border of the bright and dark regions (i.e., domains of the optimal diameters map). The limit of domains of optimal diameters and singularities in orientation maps was calculated with the 2D-gradient computation which allows to reveal pixels associated with high degree of change (panel E). As previously observed, high orientation gradient values (in yellow) were partially overlapping with high optimal diameters gradient regions (in blue).

To quantitatively estimate spatial interactions between maps, correlation coefficients between 2D-gradient of preferred orientation and optimal diameter or suppression strength maps in area 18 were computed and compared with shuffle conditions to calculate statistical confidence (see Methods). No correlation was observed between the 2D-gradient of preferred orientation and the optimal diameter [correlation: *r* = 0.02 ± 0.10, shuffle: *r* = 0.08 ± 0.10, *p* = 0.125 (*n* = 16)] or suppression strength [correlation: *r* = 0.05 ± 0.13, shuffle: *r* = 0.00 ± 0.11, *p* = 0.200 (*n* = 16)]. Panel F displays the distribution of correlation values between 2D-gradient of preferred orientation and optimal diameter in 16 independent experiments (in red) and in a shuffle condition (in green). The absence of a positive or negative correlation indicates that the spatial interactions proposed in panel A2 are unlikely. The possibility that spatial interactions correspond to the ones displayed in panels A1,A3 is tested thereafter.

The correlation coefficients between 2D-gradient of preferred orientation and 2D-gradient of optimal diameter or 2D-gradient of suppression strength maps were also calculated. A significant positive correlation was observed with 2D-gradient of optimal diameter [correlation: *r* = 0.16 ± 0.07, shuffle: *r* = 0.10 ± 0.08, *p* = 0.018 (*n* = 16)] but not with 2D-gradient of suppression strength maps [correlation: *r* = 0.21 ± 0.07, shuffle: *r* = 0.18 ± 0.11, *p* = 0.346 (*n* = 16)]. The panel G displays the distribution of correlation values between 2D-gradient of preferred orientation and 2D-gradient of optimal diameter. The absence of a negative correlation goes against the hypothesis of uniformity of coverage (panel A1). In contrast, the weak positive relationship between 2D-gradients of preferred orientation and optimal diameter maps is in accordance with the hypothesis of the spatial interaction displayed in panel A3.

## Discussion

This study presents the first evidence of the existence of surround suppression maps in the cat primary visual cortex. Orientation maps in restricted cortical regions of some millimeters (i.e., “cortical response field”) could be revealed by presenting drifting gratings in circumscribed zones of the visual field. The amplitude of the orientation response was measured in each pixel of the cortical response field at different stimulus size to determine optimal diameter and suppression strength. In three-quarters of the pixels, typical surround suppression profiles were observed, while in the remaining ones, there was no suppression. Optimal diameters were smaller in area 17 than in 18 in accordance with electrophysiological data on receptive field size of neurons in these two areas (Tusa et al., 1978, 1979). A weak relationship between optimal diameter and eccentricity was noted. No difference in suppression strengths was observed between the two cortical areas. Finally, no definite relationship between surround and orientation maps was found, with the exception that pinwheels were more likely to be present in transition zones between high and low surround suppression domains.

### Technical considerations and functional origin of the surround suppression maps

The use of a flat screen for visual stimulations can induce some (visuotopic) inaccuracies, especially at high eccentricities. Using such a design, peripheral stimuli are perceived smaller and with a higher spatial frequency than their true values. Therefore, the optimal diameters calculated here may have been overestimated because of two factors. First, the visuotopic stimulus size, including its surround space, is reduced with eccentricity. Thus, the actual surround field size contributing to suppression would also diminish with eccentricity. Second, given the eccentricity-related change in spatial frequency, the suppressive effects from the surround could have been reduced because of non-optimal stimulation of the surround field (DeAngelis et al., 1994).

Independently of the misevaluations provoked by the visual stimulation configuration, the optimal diameter values were larger than the receptive field sizes of underlying neurons (Tusa et al., 1978, 1979). Several explanations can be proposed to explain this discrepancy. As described in Dumoulin and Wandell (2008) and in Vanni et al. (2010b), in optical imaging, one pixel represents the activity of several neurons [i.e., their receptive field positions are scattered (Albus, 1975)]. Moreover, in contrast to single unit electrophysiology, intrinsic signals consist of spiking and sub-threshold activity. Finally, the neuronal response is filtered by a spatiotemporal hemodynamic response function and light scattering (Polimeni et al., 2005) that spread the signal.

The subthreshold and presynaptic activity could represent a major proportion of the optical signal. However, cortical maps, generally related to spiking activity, could also exist at the level of sub threshold and presynaptic compartments (see for example: Schummers et al., 2002; Marino et al., 2005). This could explain the presence of maps for plaid-defined pattern motion in the primary visual cortex of the cat, and the absence of such maps when considering spiking activity (Schmidt et al., 2006, 2011). It is then conceivable than the surround suppression maps presented here could be present at the sub threshold and presynaptic level only, without reliable spiking activity components.

In intrinsic optical imaging, neuronal activation is filtered by the hemodynamic response that involves a combination of an increase of cerebral blood volume and oxygenation (Dunn et al., 2005). The spatial extent of these variations could affect the quantifications of amplitudes as a function of stimulus sizes because the signal recorded in each pixel is affected by surrounding activated pixels. However, in addition to this global hemodynamic response, it has been demonstrated that local variations of hemoglobin's concentration are associated to activate domains (Mayhew, 2003). These variations are very likely to correspond to the passive transfer of O_2_ from capillaries to neurons in activated columns. The band-pass spatial filtering used in this study removed the low-pass global responses while sparing columnar activations. Thus, in the present study, the contribution of the hemodynamic coupling in the overestimation of optimal diameter values has been limited. Interestingly, the recent advances made in the field of flavoprotein autofluorescence functional imaging could provide an alternate, and more specific, method to confirm the existence of this new cortical organization in the future (Husson et al., 2007; Mallik et al., 2008; Sirotin and Das, 2010).

While the majority of neurons in the cortex are excitatory, inhibitory interneurons might nonetheless contribute to the metabolic activity measured in optical imaging. These inhibitory neurons could compromise such quantifications by increasing their metabolic demand while the activity of the network does not necessarily increase (Lauritzen and Gold, 2003). However, the impact of inhibitory neurons may not be so important given that Born and collaborators showed the presence of domains of high and low suppression in area MT of monkeys using a method based on metabolic activity measures (2-deoxyglucose) (Born and Tootell, 1992; Born, 2000). Moreover, the contribution of inhibitory interneurons in the mechanisms subtending orientation selectivity does not prevent the detection of orientation maps by optical imaging. Thus, although the involvement of inhibitory interneurons could possibly affect the linearity of the responses measured by optical imaging, they should not prevent the visualization of suppression maps in the cat primary visual cortex.

Finally, since we used vector averaging of responses in our analysis (Bonhoeffer and Grinvald, 1996; Shmuel and Grinvald, 1996), it is possible that the decrease of amplitude observed in surround suppression domains corresponds to a nonspecific neuronal facilitation. Indeed, the amplitudes of the orientation vector may have been reduced because the responses to other non-preferred orientations were larger. To robustly remove low and high frequency spatial noise in our data, we used a spatial band-pass filter, which removed the DC-component of the responses (Villeneuve et al., 2009; Vanni et al., 2010a). Thus, changes in the baseline that would have affected vector averaging quantifications were eliminated. However, surround suppression maps were also calculated by using amplitudes of the Von Mises curves fitting (Swindale et al., 2003; Villeneuve et al., 2009) and were similar to those calculated from vector averaging (data not shown). Thus, the decrease of responses observed with larger stimulus diameters likely comes from a reduction of signal amplitude.

### Functional role of the surround maps

A number of modular maps have been previously revealed by optical imaging of intrinsic signals, such as orientation (Grinvald et al., 1986), direction of motion (Malonek et al., 1994; Shmuel and Grinvald, 1996; Vanni et al., 2010a), ocular dominance (Frostig et al., 1990), retinal disparity (Chen et al., 2008), spatial frequency (Issa et al., 2000), color (Lu and Roe, 2008), and optic flow (Raffi and Siegel, 2007). These maps originate principally from the arrangements of feed-forward connections during development. However, the surround maps revealed here in the primary visual cortex could originate from a spatial organization of feedbacks from higher order regions involved in center surround interactions (e.g., area MT in primates).

A classical interaction between maps follows the principle of “uniformity of coverage.” In this spatial configuration, values of each parameter are uniformly represented in each portion of the visual field (Swindale, 1998, 2000, 2001). However, our results suggest that this organization does not prevail between surround and orientation maps. Another possible configuration of maps would locate surround domains at pinwheel sites. Indeed, the center-surround interactions could be more represented in pinwheels because orientation inputs are heterogeneous and jumps in receptive field positions are frequently found (Das and Gilbert, 1997). Therefore, the heterogenic environment of the input (i.e., positions and orientations) may be at the origin of higher suppression computations (Das and Gilbert, 1999). However, the analysis of interactions between orientation and surround maps based on the present data suggests that this kind of interaction is not encountered. This is consistent with a recent study showing that, while surround suppression is more selective to the surround orientation in domains than pinwheels, the iso-oriented suppressions were similar [see the normalized responses for 0° of relative surround orientation in the Figure 5A of Hashemi-Nezhad and Lyon (2012)]. Conversely, our study shows that pinwheels were slightly more located at the border between domains of high and low surround modulation.

The organization of neurons in functional modules involved in the analysis of a given aspect of the visual image is considered to be a strategy to optimize processing. Indeed, the clustering of neurons with comparable properties could increase the network efficiency by limiting the extent of the lateral connections (Durbin and Mitchison, 1990; Koulakov and Chklovskii, 2001; Chklovskii and Koulakov, 2004), thus reducing brain size and energetic expense. A large variety of cortical organizations is observed between homologous areas of different species and the presence of a cortical organization for one parameter could involve that the processing of this parameter is optimized in the area of this specific species (see for review: Van Hooser, 2007). For example, a cortical organization for the direction exist in the extrastriate area MT of primates (Albright et al., 1984; Malonek et al., 1994; Xu et al., 2004; Kaskan et al., 2010), an area strongly involved in motion analysis and part of the dorsal stream network (i.e., analysis of the “where,” Ungerleider and Mishkin, 1982). In contrast, no cortical organization for the direction was found in the area 21a of cat (Villeneuve et al., 2009), an area more involved in shape analysis and part of the ventral stream network (i.e., analysis of the “what,” Lomber, 2001). Interestingly, in the primate homologue of area 21a (area V4), an organization for color—a major aspect of the ventral stream—was recently revealed (Tanigawa et al., 2010).

However, the presence of ocular preference in neurons from areas not organized in ocular dominance maps raised the question of the benefit of function maps in neuronal selectivity (Drager, 1975; Weber et al., 1977; Hollander and Halbig, 1980; Adams and Horton, 2003, 2006). Alike, comparable orientation selectivity was found in V1 neurons from animals with and without orientation maps (Tiao and Blakemore, 1976; Murphy and Berman, 1979; Blasdel and Salama, 1986; Grinvald et al., 1986; Metin et al., 1988; Bosking et al., 1997; Girman et al., 1999; Ohki et al., 2005; Van Hooser et al., 2005). Noteworthy is the fact that animals without orientation maps (e.g., rodents) use vision primarily to detect predators. In contrast, primates and carnivores have orientation maps in a number of their cortical areas and use vision predominantly in behaviors such as food seeking. Although this question is still under debate, we support the hypothesis that the presence of a cortical map for a given parameter demonstrates that the computation of this parameter is optimized in this cortical area.

Hence, in cats, the suppression maps presented in this study could result from an evolutionary strategy to optimize figure ground discrimination in the primary visual cortex. This psychophysical aspect of perception uses the center/surround modulation at the cellular level and is very important in the ability to discriminate static or dynamic objects in the background, especially when the saliency is low. In the future, it would be interesting to look for suppression modules in the posterior medial bank of the Lateral Suprasylvian cortex (PMLS), considered as the cat homologue of area MT, where suppression modules have been found (Born and Tootell, 1992; Born, 2000). An interesting question is whether these surround suppression maps exist in the primary visual cortex of primates? At present time, there is no such evidence (Born and Tootell, 1992; Born, 2000) but it is possible that Born and collaborators failed to reveal a spatial organization of surround suppression in the primate area V1 because the stimulus used was optimized for MT neurons.

Interestingly, this functional cortical specialization for surround suppression would resemble the situation prevailing for motion decoding: in primates, direction modules are present in area MT but not in area V1 while they are present early in the primary visual cortex of carnivores. The hunting behavior of cats could explain this functional organization for direction and suppression at a low cortical level in order to optimize the reaction time. In primate, cortical organizations for suppression and direction look to be set aside in favor of other, more useful parameters such as color (Lu and Roe, 2008).

In conclusion, our data present clear evidence of the existence of surround modules in the primary visual cortex of the cat. This spatial arrangement at the entry level of the cortical visual system may represent a strategy to optimize figure ground discrimination in hunting animals such as carnivores.

### Conflict of interest statement

The authors declare that the research was conducted in the absence of any commercial or financial relationships that could be construed as a potential conflict of interest.
